# A systematic review on automated clinical depression diagnosis

**DOI:** 10.1038/s44184-023-00040-z

**Published:** 2023-11-20

**Authors:** Kaining Mao, Yuqi Wu, Jie Chen

**Affiliations:** Department of Electrical and Computer Engineering, University of Alberta, Edmonton, AB T6G 2R3 Canada

**Keywords:** Psychiatric disorders, Biomedical engineering, Electrical and electronic engineering

## Abstract

Assessing mental health disorders and determining treatment can be difficult for a number of reasons, including access to healthcare providers. Assessments and treatments may not be continuous and can be limited by the unpredictable nature of psychiatric symptoms. Machine-learning models using data collected in a clinical setting can improve diagnosis and treatment. Studies have used speech, text, and facial expression analysis to identify depression. Still, more research is needed to address challenges such as the need for multimodality machine-learning models for clinical use. We conducted a review of studies from the past decade that utilized speech, text, and facial expression analysis to detect depression, as defined by the Diagnostic and Statistical Manual of Mental Disorders (DSM-5), using the Preferred Reporting Items for Systematic Reviews and Meta-Analysis (PRISMA) guideline. We provide information on the number of participants, techniques used to assess clinical outcomes, speech-eliciting tasks, machine-learning algorithms, metrics, and other important discoveries for each study. A total of 544 studies were examined, 264 of which satisfied the inclusion criteria. A database has been created containing the query results and a summary of how different features are used to detect depression. While machine learning shows its potential to enhance mental health disorder evaluations, some obstacles must be overcome, especially the requirement for more transparent machine-learning models for clinical purposes. Considering the variety of datasets, feature extraction techniques, and metrics used in this field, guidelines have been provided to collect data and train machine-learning models to guarantee reproducibility and generalizability across different contexts.

## Introduction

Major depressive disorder is one of the most common diseases globally. It is estimated that 3.8% of the population is impacted by depression, and ~280 million people suffer from depression. The economic impact of depression is significant, even when compared to other medical conditions, such as cancer, cardiovascular diseases, diabetes, and respiratory diseases^1^. Conventional methods for assessing and monitoring depression involve semi-structured interviews between patients and healthcare workers, which can be subjective and affected by bias (i.e., deemphasized or exaggerated symptoms), cognitive limitations (i.e., memory errors) and social stigma. The need for objective depression diagnosis, routine symptom monitoring, and timely treatment is widely recognized in the medical community. However, many people with depression have difficulty accessing psychological healthcare services due to geographical and financial barriers. As the World Health Organization (WHO) has reported, over 75% of individuals with depression in low and mid-income countries do not receive qualified psychotherapy^2^. The shortage of well-trained healthcare professionals and the social stigma surrounding depression are major barriers patients face when seeking help. An automated depression assessment tool would assist in objective diagnosis and remote care to improve the quality of mental healthcare.

One potential solution to improve the objectivity of depression assessments and the quality of mental health services is to enhance mental health-related data collection and analysis via sensors and advanced machine-learning algorithms. Sensors that measure biological signals such as electrocardiogram, electroencephalogram, heart rate, and skin conductance may also be used to monitor biomarkers of depression^3–15^. Researchers have developed models to detect depression and other psychological disorders by extracting features from interview videos, or audio recordings^16–28^. Toolkits such as OpenFace can extract facial landmarks, action units, face orientation, and eye gaze^29^. Other modalities, such as neuroimaging data, have been used to predict the presence of Schizophrenia^30^. Still, here we will focus only on non-neuroimaging modalities, such as acoustic, semantic, and facial modalities. Lastly, features extracted from social media and transcribed audio recordings have also been used to detect depression and stress^31,32^. These previous studies provide valuable insights into new approaches for improved depression assessment.

A machine-learning algorithm for automated depression assessment can provide several benefits, including (a) supporting clinicians in making accurate diagnoses and providing effective treatment; (b) identifying individuals at risk for depression before they seek treatment from mental healthcare clinics and (c) tracking symptoms over time, both during and after treatment. These benefits are further discussed in the following:

One of the primary advantages of automated depression assessment is that it can help overcome barriers preventing people from receiving proper diagnoses and treatment for mental health issues in a timely manner. Mohr et al. reported that the main barriers patients face when seeking help are stigma, lack of motivation, time or availability constraints, and cost^33–35^. Automated diagnostic tools could allow depression patients who have not yet sought help from healthcare professionals to evaluate their mental states remotely and receive online support from healthcare workers. In addition, these tools can be designed to customize treatment based on an individual’s specific symptoms, improving treatment efficacy^36,37^. These systems can also be utilized for mental disorder screening in various settings, such as universities, the military, and basic healthcare facilities.

Automated depression assessment systems can play an important role in helping doctors diagnose and make decisions related to depression. Diagnosing depression can be difficult because symptoms may be episodic and multiple disorders may occur simultaneously, as demonstrated by the low inter-rater reliability^38^ and test–retest reliability scores^39^ in major depressive disorder diagnosis. In addition, it is also challenging to train a model to recognize any mental disorder, given that patients may have multiple disorders that present overlapping symptoms simultaneously. For example, over 50% of depression or anxiety cases co-occur with drug abuse or post-traumatic stress disorder (PTSD)^40^. Because of these challenges, researchers have proposed prediction tools for suicidal thoughts and behaviors across many mental disorders (for a review, see ref. ^41^). The Research Domain Criteria, created by the National Institute of Mental Health, assists in distinguishing diagnoses and symptoms^42^. Consequently, we can train models to predict the probability of different mental disorders to assist in both diagnosis and early interventions. Furthermore, prediction tools can enable customized treatment plans based on multimodality features (genetic, behavioral, neuroimaging)^43–45^. As a result, automated depression assessment models improve the efficiency of the healthcare systems, lower costs, and make treatment plans more customizable.

Automated depression assessment models can also enhance mental healthcare by allowing more frequent monitoring of symptoms, even in real time. Real-time monitoring enables individuals at risk for depression to be reminded to seek mental healthcare. Additionally, depression symptoms can change between healthcare appointments^46^. Real-time monitoring can detect important signs related to suicidal or self-harm thoughts and conduct online psychotherapy. With real-time monitoring, patients and clinicians can monitor symptoms, conduct early intervention, and tailor treatment plans in a more personalized and timely manner.

Despite the potential benefits, these advantages have yet to be fully realized. Previous studies have relied on small, unrepresentative and non-clinical datasets, which are inadequate as they do not reflect real-world clinical situations. Most clinical datasets are limited in size and data type, for example, recorded in noise-controlled rooms, spoken in English, and restricted to adult participants. Moreover, models can be biased toward differences in the demographic characteristics of patients and healthy controls. For example, the model may tend to assign lower depression likelihood in male subjects because fewer male subjects have depression in the training set^47–51^. Another significant issue in developing high-performance models is that their output depression probabilities are difficult to explain, limiting their clinical applications. This is because machine-learning models are often considered “black boxes”, meaning that their decision-making process is not easily interpretable by humans. While high-performing models can achieve high accuracy in depression prediction, the lack of transparency in how they made such decisions can be a barrier to clinical use. Thus, an explanation of the output probabilities of high-performance models is crucial for developing interpretable automated depression assessment models.

The study of speech patterns has been a research topic in identifying indicators of mental disorders since the 1920s. Emil Kraepelin, the founder of modern scientific psychiatry, reported that the voices of depressed patients were lower in pitch, sound intensity, and speech rate, and instead tend to be monotonous and hesitant, with shuttering and whispering^52^. Moreover, acoustic features can be extracted across different languages, which is important for languages without pre-trained natural language processing models. In addition, speech recordings can be easily collected with smartphones and laptop computers instead of complex and costly equipment. With the advancements in speech recognition, especially its application for electronic medical records, speech recording will become more accessible for research purposes.

In research settings, depression is conventionally assessed using clinical depression rating scales. However, in clinical practice, the official psychiatric diagnosis is typically determined through a clinical interview, which may be semi-structured, with rating scales used as supplementary data to inform the diagnosis. However, these rating scales have limitations, as responses can be influenced by factors such as the patient’s emotional state, relationship with the clinician, and patient self-bias (e.g., participants may be more likely to exaggerate their symptoms)^53^. With the advancement of machine-learning applied to text data from social media, new methods have emerged to address these limitations. Social media such as Twitter, Facebook, and Reddit provide a wealth of information about individuals’ feelings, thoughts and activities. Machine learning, especially text mining and sentiment analysis techniques, have become more accurate and intelligent, aiding mental healthcare providers in detecting depression. For instance, Pirina et al. and Yates et al. proposed machine-learning models to screen depression symptoms based on text data from Reddit^54,55^. Researchers have recently developed models that utilize semantic features and other information extracted from social media platforms for detecting depression^56–60^.

Several studies have been published on depression detection via facial image processing. The Audio/Visual Emotion Challenge and Workshop (AVEC) depression sub-challenge published papers with encouraging results on depression detection. Jan et al. achieved plausible results with a Motion History Histogram to extract Local Binary Pattern (LBP) and Edge Orientation Histogram (EOH) for depression identification^61^. Other features, such as head posture, blink rate, and eye gaze, are also reported to be effective in depression detection^62,63^. Depressive individuals tend to display less nodding, avoid eye contact, and lower their heads more frequently than healthy individuals. Alghowinem et al. developed a support vector machine (SVM) to recognize depression and concluded that the head movements of depressed individuals are different from those of healthy controls^64^.

This paper reviews recent research on the use of computational methods to predict major depressive disorder using acoustic, semantic, and facial features. This review is unique in that it uses the Preferred Reporting Items for Systematic Reviews and Meta-Analysis (PRISMA) guidelines for extensive and rigorous evaluation of the latest research findings. By synthesizing and analyzing recently published data, this review offers important insights into the current state of the field and identifies key areas for future research. We would like to show how multimodal features could enhance mental healthcare, which provides insights into establishing connections between psychiatry and computing. Consequently, this review aims to (a) summarize results from previous publications using acoustic, semantic, and facial features to detect major depressive disorder; (b) characterize psychiatric disorders by identifying significant differences in acoustic, semantic, and visual features; (c) associate these multimodal features to depression symptoms and behaviors; and (d) summarize the challenges of automated depression assessment, make suggestions for future data collection and model training with better reproducibility and performance. Therefore, we propose that artificial intelligence may be a key tool for improving the assessment and treatment of depression through automated approaches.

## Methods

### Inclusion criteria and literature search

**Fig. 1:**
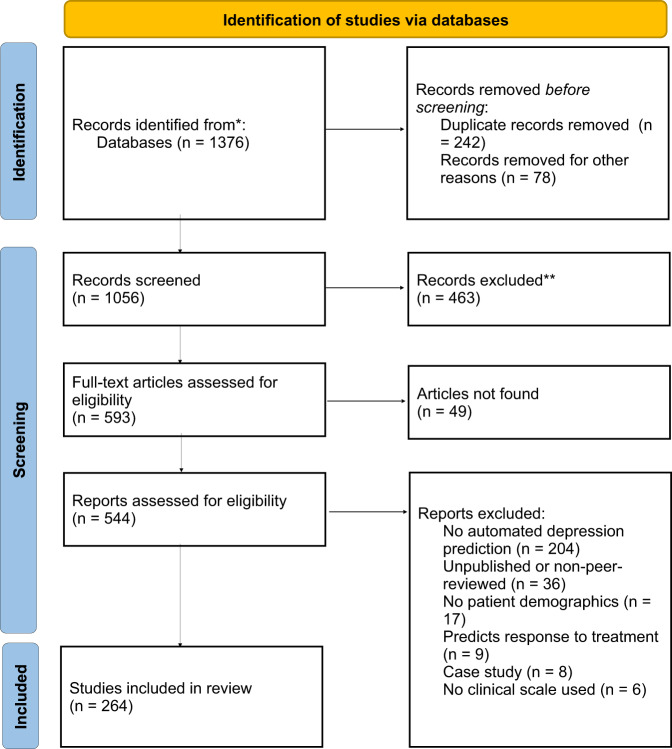
PRISMA flow diagram of study inclusion and exclusion criteria in this review.

The PRISMA guidelines were followed in this literature review, as shown in Fig. 1. Our goal was to search for articles published in the last ten years that included artificial intelligence methods for predicting the presence or severity of major depressive disorder by analyzing acoustic, semantic, and facial landmarks. Google Scholar was used as the search engine for articles from 2012 to the present, queried between July 20, 2022, and May 20, 2023, excluding case studies, studies that solely used perceptual evaluation of speech, studies without a control group or clinical depression rating scales, non-peer-reviewed preprint and theses, and articles published before 2022 and having fewer citations than years of publication (e.g., articles published in 2019 with three citations, or articles published in 2017 with five citations would be included). We excluded certain articles that lacked comprehensive methodology or detailed results. In addition, we encountered cases where articles were published in both journals and conference proceedings, covering similar topics, methods, and results. Furthermore, some articles only focused on proposing methods for feature extraction without incorporating the training of models for depression detection. The search terms used to find relevant articles were: “allintitle: ((“depression” OR “major depressive disorder”) + (acoustic OR acoustical OR speech OR voice OR vocal OR audio OR pitch OR prosody OR prosodic OR vowel) + (automated OR behavioral OR measures OR diagnosis)).” Articles related to depression caused by Parkinson’s Disease, autism, and substance overdose disorders were excluded. Replacement of the acoustic feature with the semantic feature and facial landmarks in the command resulted in the following search term with associated features: “allintitle: ((“depression” OR “major depressive disorder”) + (semantic OR text OR interview OR transcript OR social media) + (automated OR behavioral OR measures OR diagnosis))” and “allintitle: ((“depression” OR “major depressive disorder”) + (facial expression OR visual OR facial features OR facial landmarks OR facial muscles)+ (automated OR behavioral OR measures OR diagnosis)).”

Information extraction was performed by reading the title, abstract, and conclusion. The following information was synthesized from each article: mental disorders, number of subjects, age range, optimal model, best metrics, type of validation, and predictive features.

### Reporting summary

Further information on research design is available in the Nature Research Reporting Summary linked to this article.

## Results

### Summary of results

In total, 264 studies were included in the review. Table 1 summarizes the search results. Synthesized information can be found online https://bit.ly/3DBQtZk, https://bit.ly/43Q6Yvy, and https://bit.ly/44IKaPv, which can be extended by adding new studies on a blank row or fields on a blank column. Previous review and datasets-only articles were included in this study but are not included in Table 1.

**Table 1:** Summary of literature review results.

Modality	Articles	Median dataset size (range)	Clinical assessment	Predictive models
Acoustic	140	189	36	140
Semantic	99	1046	2	81
Facial landmarks	25	49	16	21

### Predictive acoustic features in major depressive disorder

**Fig. 2:**
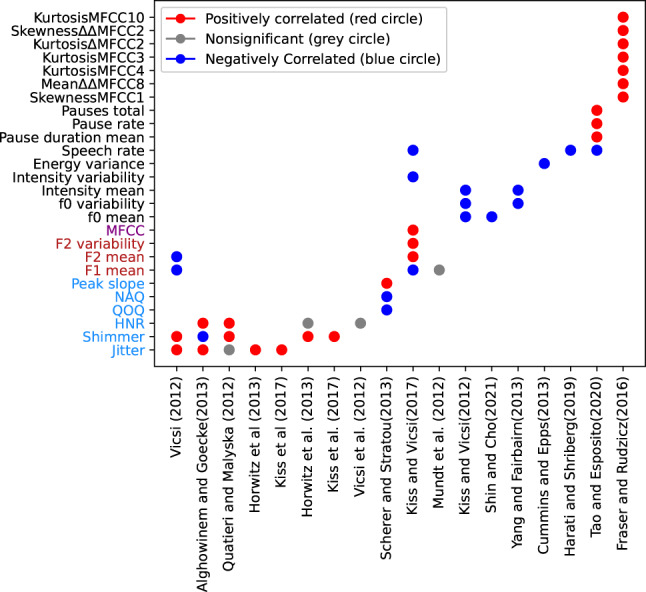
Synthesis of acoustic feature analysis in major depressive disorder. Acoustic features are sorted, such as vocal fold source features (blue), vocal tract filter features (red), spectral features (purple), and features related to prosody or melody (black). Features that are significantly higher in a psychiatric group than healthy controls or that correlate positively with the depression level receive a score of 1 (red), features that are lower or correlate negatively receive a score of -1 (blue), and nonsignificant or contradicting findings receive a score of 0 (gray). Features not studied in any studies are blank.

With the publicly available code provided by Low et al.^65^, we created Fig. 2, which provides a synthesis of the acoustic features investigated using machine learning. The table shows the acoustic features found to be statistically different between the group with a mental disorder and the healthy control group or highly correlated with a diagnostic rating scale. Each cell in Fig. 2 represents the correlation between a specific acoustic feature and depression. For example, an acoustic feature that correlates positively with the disorder severity would be marked with a red dot, a negative correlation with a blue dot, and a nonsignificant feature with a gray dot.

Table 2 provides an overview of the key findings from previous studies on automated depression detection using acoustic features. One common finding among the studies is the relationship between acoustic volume and depression. Cummins et al.^66,67^ found that as the level of depression increases, the acoustic volume significantly decreases, indicating a potential acoustic marker for depression. Similarly, Harati et al.^68^ reported that individuals with depression tend to have lower voices compared to the control group.

**Table 2:** Predictive acoustic features in prior research publications.

Study	Key finding
Cummins et al.^66^	Decreased acoustic volume
	More concentrated MFCC space
Cummins et al.^69^	Gender-dependent formant features
Morales et al.^70^	Fundamental frequency
	Pronoun use and negatively valenced words
Scherer et al.^118^	Tenser voice
Vicsi et al.^71^	Jitter and shimmer values of vowels
	First and second formant frequencies
Marmor et al.^150^	Seeking care for voice problem
Cummins et al.^67^	Acoustic volume
	Probabilistic acoustic volume slope
Harati et al.^68^	Lower voices
	Variance in voice pitch
Kiss et al.^72^	Articulation rate
	Speech rate
	Pause lengths
	Formant frequency
Stasak et al.^73^	Use phonemes that require less effort
	Articulatory precision

Another notable finding is the influence of gender on acoustic features related to depression. Cummins et al.^69^ proposed gender-dependent formant features that outperformed acoustic-only features in depression detection. This suggests that gender-specific acoustic characteristics may play a role in accurately detecting depression. Fundamental frequency (*F*_0_) was found to be negatively correlated with depression level by Morales et al.^70^. This finding indicates that changes in *F*_0_ could serve as an indicator of depression. In addition, Vicsi et al.^71^ discovered that depressed individuals exhibited higher jitter and shimmer values in vowel production, along with lower first and second formant frequencies. These acoustic features may serve as potential biomarkers for depression detection. Kiss et al.^72^ highlighted the importance of speech rate, articulation rate, pause lengths, and formant frequency in detecting depression. They found that these acoustic features differed between individuals with depression and the control group, suggesting potential utility in automated depression detection. Stasak et al.^73^ proposed that depressed individuals tend to use phonemes that require less effort and demonstrate decreased articulatory precision. These findings indicate that analyzing articulatory characteristics could provide valuable insights for depression detection.

It is important to note that these findings are based on previous studies and should be interpreted within the context of their respective methodologies and limitations. Further research is needed to validate and refine the use of these acoustic features for automated depression detection.

### Predictive semantic features in major depressive disorder

This summary reviews studies on automated depression detection using language cues. Previous studies identified depression based on clinician diagnosis, patient self-reported mental status and online forum memberships. Clinician diagnosis means that depression levels were determined by a clinician based on interview transcripts or online posts. Among the 99 studies using language cues, only two identified depression based on the clinician diagnosis^74,75^, while 77 studies used self-reported depression rating scales. The remaining 20 studies did not report which criteria were used to determine depression levels.

#### Social media and depression

Table 3 provides a summary of key findings from various studies examining the relationship between social media usage and depression. These studies employ a range of techniques and models to improve the detection and understanding of depression based on social media data. Several studies highlight increased social media usage among individuals with depression. Various advanced models, such as GRU models with knowledge-aware dot-product attention^76^ and DeepBoSE^77^, demonstrate improved performance in depression detection compared to conventional methods. In addition, semantic mapping of emoticons^78^ and the application of semantic role labeling^79^ are proposed as techniques to enhance detection accuracy. These findings highlight the potential of leveraging machine learning and natural language processing techniques to gain insights into mental health conditions through social media data.

**Table 3:** Exploring the predictive relationship between social media usage and depression.

Author	Main findings
Hartanto et al.^151^	Social media usage increases among depressive individuals
Figueredo et al.^78^	Semantic mapping of emoticons improves the performance.
Stankevich et al.^79^	Future work needs to involve applying semantic role labeling to obtain better results.
Lara et al.^77^	DeepBoSE outperforms conventional Bag-of-Features(BoF) representations.
Hussain et al.^152^	Proposed depression lexicons that distinguish depressive individuals.
Ramiandrisoa et al.^86^	Analyzing users’ social signals could be considered for further analysis.
Liaw et al.^153^	Topic modeling features such as liked tweets can be useful.
Guo et al.^84^	Fused the lexical features using a correlation-based metric to enhance prediction effectiveness.
Cui et al.^83^	Capture deep emotional information from the input embeddings with a pre-trained TextCNN.
Zogan et al.^87^	The model captures semantic features from user timelines for depression detection.
Tlatelpa et al.^80^	User characteristics and sentiment analysis improved depression detection performance.
Cha et al.^82^	Proposed lexicon features for depression detection.
Primack et al.^154^	Using multiple social media platforms is associated with depression.
Primack et al.^155^	Social media use is associated with the development of depression.
Vedula et al.^156^	Depressed users exhibit reduced online activities, increased negative sentiment, and self-focused pronoun usage.
Nesi et al.^157^	More frequent negative emotional reactions to social media are linked to more severe depression symptoms, especially among female subjects.
Thorisdottir et al.^158^	Time spent on social media has a stronger relationship with emotional distress among female subjects.
Ghosh et al.^159^	Depressed users frequently use negative words and mostly post late at night, in addition to increased use of personal pronouns and sharing personal events.
Aragon et al.^160^	Representations based on fine-grained emotions can more comprehensively capture users experiencing depression.
Puukko et al.^161^	Depressed individuals increasingly use active social media during early and late adolescence.
Robinson et al.^162^	Depressed individuals are more likely to compare themselves to others and dislike being tagged in self-perceived unflattering pictures.
Choudhury et al.^163^	Social media can provide valuable indicators of depression onset, including decreased social activities, increased negative emotions, focus on personal and medical issues, and more frequent expressions of religious involvement.

Furthermore, the studies presented in the table emphasize the importance of considering multimodal data, user characteristics, and sentiment analysis for a comprehensive understanding of depression^80,81^. They also propose the use of lexicon features and emotional information capture to improve depression detection^82,83^. The fusion of lexical features and the development of bipolar feature vectors demonstrate promising results in enhancing prediction effectiveness^84,85^. In addition, the studies suggest the potential of analyzing social signals and user timelines to capture semantic features for depression detection^86,87^.

It is important to note that while these studies offer valuable insights, there are still ethical considerations that need to be addressed. Responsible data usage, privacy protection, and potential biases are crucial aspects that should be carefully considered when developing AI-based depression detection tools using social media data. Furthermore, future research should focus on the validation and generalization of these findings across diverse populations and cultures.

**Fig. 3:**
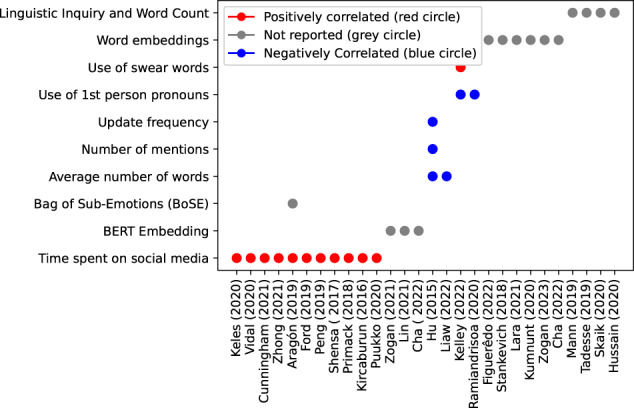
Synthesis of semantic feature analysis in major depressive disorder. Features that are significantly higher in a psychiatric group than healthy controls or that correlate positively with the depression level receive a score of 1 (red), features that are lower or correlate negatively receive a score of −1 (blue), and findings without reporting their changes receive a score of 0 (gray). Features not studied in any studies are blank.

By synthesizing these findings (see Fig. 3), we have gained a deeper understanding of the potential of social media data in detecting and understanding depression. These insights can inform the development of effective mental health interventions, improve clinical practice, and contribute to the responsible and ethical usage of AI in this domain.

#### Problematic social media use

Table 4 provides a summary of the main findings from studies examining the association between problematic social media use and depression, including several studies^88–90^ which consistently found a strong correlation. In addition, findings from refs. ^91–93^ indicate that over-sharing and stressed posting on social media is associated with depression. These findings collectively highlight the importance of recognizing problematic social media use as a potential risk factor for depressive symptoms. The results underscore the need for interventions and guidelines to promote healthier social media habits, particularly in vulnerable populations such as adolescents and university students. It is important to acknowledge the limitations of the studies summarized in Table 4, including factors like sample size, study design, and generalizability. Future research should consider longitudinal studies to examine the long-term effects of excessive social media use on depression and explore the underlying mechanisms of this association.

**Table 4:** The impact of problematic social media use on mental health.

Author	Main findings
Cunningham et al.^88^	Future research should focus on individuals with problematic social media use.
Shensa et al.^89^	Problematic social media use is strongly associated with depressive symptoms.
Woods et al.^90^	Adolescents who abused social media have a higher depression risk.
Radovic et al.^91^	Over-sharing and stressed posting is associated with depression.
Ivie et al.^92^	Significant positive correlation between social media use and depressive symptoms.
Raudsepp et al.^93^	Excessive social media use was correlated with increased symptoms of depression.
Zhong et al.^164^	Breaks from social media use could have mitigated mental health trauma during the pandemic.
Haand et al.^165^	Addiction to social media is positively linked to depression.
Brailovskaia et al.^166^	Depressed individuals often intensively use social media to escape negative moods.
Jeri-Yabar et al.^167^	Excessive use of social media is associated with depressive symptoms.
Kircaburun et al.^168^	Social media addiction indirectly affects depression levels.

Overall, the findings contribute to our understanding of the complex relationship between social media use and depression, providing valuable insights for mental health promotion and clinical practice.

#### Machine-learning models

Automated depression detection algorithms have been a subject of study by various researchers. Table 5 provides a summary of these studies, highlighting machine-learning models and performance metrics employed. Salas et al. conducted a comprehensive review of previous studies that utilized language cues and found that word embedding was the most commonly used linguistic feature extraction method, while the support vector machine was the most prominent machine-learning model^94^. Liu et al. summarized the findings of studies focused on using machine-learning methods to detect depressive symptoms in social media data, highlighting their potential as complementary tools in public mental health practice^95^. Yazdavar et al. developed a semi-supervised statistical model to assess the alignment between depressive symptoms expressed on Twitter and medical findings reported via the Patient Health Questionnaire (PHQ-9), achieving 68% accuracy and 72% precision in identifying clinical depressive symptoms^74^. Zogan et al. introduced a computational approach for automatically identifying depression using a hybrid extractive summarization technique applied to tweets, achieving high recall, precision, and *F*_1_ score^75^. Aragón et al. proposed a novel representation called bag of sub-emotions (BoSE) that improved the detection of depression using fine-grained emotions, demonstrating competitive results compared to existing approaches^57^. These studies collectively emphasize the significance of linguistic features, word embeddings, and machine-learning models in automated depression detection.

**Table 5:** Analyzing the efficacy of machine-learning models in detecting depression through social media data.

Author	Main findings
Yazdavar et al.^74^	Achieved 68% accuracy and 72% precision in identifying clinical depressive symptoms using a semi-supervised statistical model.
Zogan et al.^75^	Proposed a new computational model and achieved a recall of 0.904, precision of 0.909, and F1 score of 0.912.
Aragón et al.^57^	Using fine-grained emotions to obtain competitive results in comparison to state-of-the-art approaches.
Paul et al.^32^	AdaBoost classifier outperformed other methods for depression likelihood assessment.
Ricard et al.^169^	Leveraging community-generated content from social media can be informative for automated depression assessment.
Peng et al.^170^	Demonstrated that a multi-kernel support vector machine is the most appropriate approach to identifying depression in individuals using social media.
Aldarwish et al.^171^	Trained a support vector machine based on term frequency to classify depression levels.
Chiong et al.^31^	The proposed model effectively determines depression presence via social media posts, even when the training datasets do not contain depression-related words.
Burdisso et al.^172^	Introduced a general framework for early depression detection with less computational cost and higher interpretability.
Smys et al.^173^	A machine-learning model consisting of a support vector machine and a naive Bayes model can predict depression in its early stages.
Bucur et al.^174^	Latent semantic analysis shows a significant difference in writing topics depending on users’ mental health.
Kayalvizhi et al.^175^	A word2vec pre-trained word embedding and random forest classifier achieved their best performance with a 0.877 *F*_1_ score.
Mann et al.^176^	Fusion model can detect moderate depression or higher with 0.92 recall and 0.69 precision.
Sadeque et al.^177^	Proposed a system to effectively detect depression using social media content with an accuracy of 88% and *F*_1_ score of 93%.
Hussain et al.^152^	Application accurately identifies indicators of depression in Facebook users with 94% accuracy.
Tadesse et al.^58^	Achieved 91% accuracy and *F*_1_ score of 93% with a multi-layer perceptron algorithm and combined features.
Fatima et al.^178^	Achieved an accuracy, recall, and precision of 91.7% using a combination of text-based features and machine-learning techniques.
Katchapakirin et al.^179^	Facebook behaviors can be used to predict depression levels with an accuracy of 85% and *F*_1_ score of 88.9%.
Shen et al.^180^	The model outperformed several baselines by 3% to 10% with an *F*_1_ score of 85%.
Li et al.^181^	Proposed a correlation explanation learning algorithm to detect COVID-19-related stress symptoms.
Lin et al.^182^	Social media use is significantly associated with increased depression risk.

However, some weaknesses and variations in the literature have been raised. McCrae et al. conducted a review of studies examining the relationship between social media use and depression symptoms, highlighting the need for comparative analysis due to variations in methods, sample sizes, and results across studies^96^. They also suggested that future research should incorporate longitudinal analysis, as most studies were cross-sectional. Similarly, Heffer et al. found no predictive association between social media use and depressive symptoms over time, challenging the assumption that social media use leads to depressive symptoms^97^. These contrasting perspectives call for further investigation and highlight the complexity of the relationship between social media use and depression.

In conclusion, while automated depression detection algorithms show promise, the field still faces challenges in terms of standardization, methodological variations, and the need for longitudinal analysis. Future research should address these limitations, conduct a comparative analysis, and explore the intricate mechanisms underlying the relationship between social media use and depression. In addition, ethical considerations and the potential impact of using social media data for mental health assessment should be carefully examined. Advancements in this field can contribute to the development of effective and reliable tools for early detection and intervention in depression.

#### Privacy issues and other factors

Ford et al. investigated social media users’ opinions on providing mental healthcare services for depression-vulnerable individuals by analyzing social media content. Their survey indicated that social media users post negative content during low moods. They could see the benefits of identifying depression using social media content but did not believe that the risks of privacy breaches outweighed these benefits. In this survey, most participants consider identifying depression symptoms using social media content is intrusive and would not grant permission to researchers to conduct linguistic analysis^98^.

Gender also affects text patterns and must be accounted for when developing depression detection models. Hou et al. investigated the gender differences in depression and explored associated factors during the COVID-19 pandemic among Chinese social media users. Their findings showed an increased prevalence of depression and anxiety in the Chinese population, with females more likely to experience more severe symptoms than males^99^.

Several studies have built text corpora from social media, which were used to train baseline datasets, thus allowing other researchers to develop more efficient prediction models. Choudhury et al. built a large collection of tweets from individuals clinically diagnosed with depression. They developed a support vector machine with a radial basis function kernel to characterize depression levels in populations^100^. Narynov et al. presented a dataset collected from social network platforms that are commonly used by the youth of the Commonwealth of Independent countries. They demonstrated that the dataset has high validity and can be used for further research in mental health^101^. These studies contribute to our understanding of social media as a tool for mental health analysis and the importance of gender differences in depression research.

### Predictive facial features in major depressive disorder

Table 6 provides a summary of previous studies on automated depression detection using facial features. The studies examined various aspects of facial expressions and their relationship to depression. Among the 18 studies that utilized facial landmark features, 13 studies identified depression based on clinician diagnosis, while four studies used self-report depression rating scales. One study did not report the specific criterion used to determine depression levels.

**Table 6:** Exploring the predictive relationship between facial expressions and depression.

Author	Main findings
Li et al.^102^	A deep residual regression model to evaluate depression levels using enhancement techniques can reduce the influence of external factors on the image, significantly improving prediction performance.
Wang et al.^62^	Facial analysis is effective in automated depression diagnosis with an accuracy of 78%, recall of 80%, and F1 score of 79%.
Hao et al.^103^	A bidirectional LSTM network with an attention mechanism achieved an accuracy of 82% and F1 score of 81%.
Hunter et al.^63^	Individuals with depressive symptomatology showed a different eye-tracking pattern in processing emotional expressions.
Jan et al.^61^	The linear regression method applied to the AVEC 2014 dataset can predict BDI score using natural facial expressions.
Mohan et al.^183^	The proposed LSTM had the highest accuracy compared to other baselines.
Lee et al.^184^	An accessible depression diagnosis system using real-time object recognition and facial expressions obtained with a smartphone camera.
Liu et al.^104^	Proposed Part-and-Relation Attention Network for depression recognition, which outperforms state-of-the-art models with smaller prediction errors and higher stability.
Hamid et al.^105^	Designed a model for depression detection using electroencephalogram (EEG) and facial features. A hybrid model is proposed, outperforming existing diagnosis systems.
Nasir et al.^106^	A multimodal classification system for depression detection using geometrical facial features. The proposed visual feature sets show potential for robust and knowledge-driven depression classification.
Dai et al.^107^	A multimodal model with high performance on the AVEC 2013, AVEC 2014, and Emotion-Gait datasets. They concluded that the visual model is accurate.
Shangguan et al.^108^	An aggregation method which achieved comparable performance to 3D models with fewer parameters. The study suggests that video stimuli can be used for automatic depression detection.
Sumali et al.^185^	Significant differences were observed in facial landmark features (e.g., average right nose (speed), median left ear top (speed), and left pupil-right pupil positions) between healthy and depressive volunteers.
Dadiz et al.^186^	The uniformed local binary pattern extracted from videos for depression detection focuses on specific facial areas.

Li et al.^102^ proposed a deep residual regression model that evaluated depression levels. Their findings indicated that enhancing techniques can significantly improve prediction performance by reducing the influence of external factors, such as lighting and head pose. Wang et al.^62^ analyzed facial expressions in videos for automated depression diagnosis. Their study demonstrated the effectiveness of facial analysis, achieving an accuracy of 78%, recall of 80%, and an F1 score of 79%. Hao et al.^103^ investigated optimal methods of depression detection using contextual temporal information. Their proposed bidirectional long-short-term memory network (LSTM) with an attention mechanism achieved an accuracy of 82% and an F1 score of 81%. Hunter et al.^63^ evaluated the eye-tracking patterns of individuals with non-clinical depressive symptomatology in processing emotional expressions, revealing distinct differences compared to healthy individuals.

In addition to the studies mentioned in the original paragraph, several more recent studies provide valuable insights into automated depression detection using facial features. For example, Liu et al.^104^ proposed the Part-and-Relation Attention Network, which outperformed state-of-the-art models with smaller prediction errors and higher stability. Hamid et al.^105^ designed a hybrid model that integrates electroencephalogram (EEG) data and facial features, surpassing existing diagnosis systems. Nasir et al.^106^ explored multimodal classification systems using geometrical facial features, indicating potential for robust and knowledge-driven depression classification. Dai et al.^107^ proposed a multimodal model that achieved superior performance on multiple datasets, emphasizing the accuracy of the visual model. Shangguan et al.^108^ demonstrated that video stimuli and an aggregation method can be effective for automatic depression detection.

**Fig. 4:**
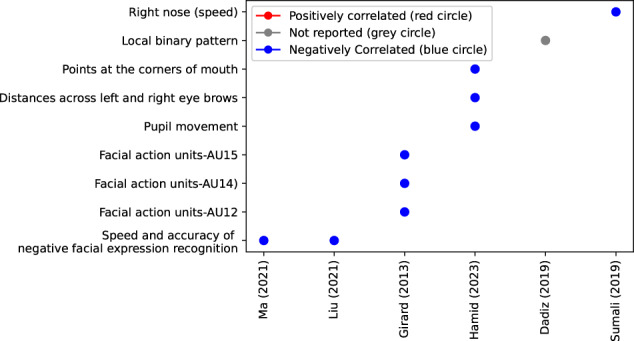
Synthesis of visual feature analysis in major depressive disorder. Features that are significantly higher in a psychiatric group than healthy controls or that correlate positively with the depression level receive a score of 1 (red), features that are lower or correlate negatively receive a score of −1 (blue), and findings without reporting their changes receive a score of 0 (gray). Features not studied in any studies are blank.

Overall, the summarized studies (see Fig. 4) highlight the significance of facial expressions in automated depression detection. They showcase various approaches, including deep learning models, multimodal techniques, and analysis of specific facial features. These findings contribute to the understanding of how facial expressions can serve as valuable indicators for detecting and diagnosing depression.

## Discussion

Most studies in this literature review adopted automated speech feature extraction to assess major depressive disorder. This is probably due to the Audio/Visual Emotion Challenge Workshop (AVEC) competitions, which provide automated extracted audio and video features to predict the severity of these conditions. Many other studies then used the public datasets in competitions like Distress Analysis Interview Corpus Wizard of Oz (DAIC-WOZ). Of the 264 studies in this review, 39% used DAIC-WOZ or AVEC datasets.

Most of the studies used some form of cross-validation for evaluating the performance of the trained models. However, only some studies used held-out test sets, which means that most models’ reported performance may not generalize well. Without a held-out test set, performance may drop from the development set to the test set, as has been observed in AVEC competitions^109–111^. In contrast, models that used held-out test sets generally performed better on the test set^17^. In addition, Zhang et al. achieved performance close to the state-of-the-art on the AVEC-2019 dataset (mean absolute error = 5.77 on the test set) by using automated extracted features and a random forest classifier^111^. This suggests that the performance of a depression detection model is dependent on the dataset size, preprocessing strategy, feature engineering, and the model itself, which are all determined by the dataset used for training. Different models applied to different datasets can lead to different complexity-accuracy tradeoffs, and there is no universal best model.

When studying the significance of acoustic features for major depressive disorder, many automated extracted features have been found to be predictive, and their correlations may be useful for making depression predictions^70,106,112,113^. As a result, we examine the connection between these automated extracted features and the observable symptoms of major depressive disorder.

### Associating acoustic features to depression symptoms

Many studies have reported that individuals with depression have lower values of the fundamental frequency *F*_0_ and its range, which indicates that their speech becomes monotonous^70,112,114^. In addition, acoustic features such as jitter and shimmer^115,116^ have been observed to increase with the severity of depression, which may be a result of slower thought, reaction, and physical movement in people with depression.

### Review for data collection

The datasets used in automated depression detection studies vary greatly in size, participants’ demographics, depression rating scales, the task used to elicit emotion, and the interview environment. Therefore, the performance of a detection model can be misleading if the dataset used for training is not representative of the studied population. In this review, we discussed the data collection strategies used in these studies to elicit emotions, record videos, and maintain participant privacy while avoiding confounding factors such as clinician questions and patient responses.

### Identifying the presence of comorbidity

Many previous studies have not reported comorbidities^117^ which presents additional challenges when developing automated depression detection models. However, Scherer et al. discovered a strong association (Pearson’s *r* > 0.8) between scores for depression on the PHQ-9 scale and scores for PTSD on the PTSD Checklist-Civilian Version (PCL-C) in the DAIC-WOZ dataset^118^. Only a few articles stated that they excluded individuals with comorbidities from their study, such as ref. ^119^. To improve the data quality through consideration of comorbidities, researchers should use multiple mental disorder rating scales when collecting data. Additionally, researchers should develop and compare models trained with and without comorbidities to better understand the impact on the model performance.

### Factors to consider in recruiting control groups

When selecting control groups for depression studies, it is important to ensure that individuals in the control group do not match any diagnostic criteria for other pathological conditions. For example, an individual may not be assessed as having depression based on their depression rating scale, but they may be assessed as suffering from PTSD that affects their speech patterns. Age, gender, first language, comorbidities, brain injury, respiratory disorders, and drug abuse can also affect speech and facial landmark patterns. In addition, variables such as education level, race, medication, and gender^120–122^ can also affect speech patterns. Hert et al. have reported that antipsychotic therapies may lead to dyskinesia, an involuntary movement of facial muscles that affects speech and facial landmarks^123^. Therefore, individuals with a history of antidepressant medication should be excluded or reported in depression detection studies. Other variables such as gender, age, and education level can be adjusted via propensity score matching if they are statistically different between the depressive and healthy control groups.

### Self-report depression rating scales: pros and cons in depression diagnosis

The traditional method for diagnosing depression is via a clinical evaluation by a registered psychologist, which is considered the gold standard compared to self-report depression rating scales. However, clinical diagnosis can be costly and subject to the experience and expertise of the clinician, leading to lower inter-rater reliability^38^. Most studies included in this review relied on self-reporting depression rating scales instead of clinical evaluation, such as AVEC^121,122^ and DAIC-WOZ^120^. When using these self-rating scales, the task becomes predicting the self-report rating scale rather than a clinical diagnosis, which may not align with a clinician’s evaluation. On the other hand, using open-source datasets for research can improve reproducibility and objectively compare model performance.

### Eliciting emotions in depression diagnosis

Choosing appropriate tasks for eliciting emotions is crucial, as specific features may be linked to certain depression rating scales but not others. In Table 7, we summarize the tasks used in previous articles and their advantages. Kane et al. proposed that sustained vowels are optimal for estimating glottal source features because it can be difficult to identify voiced sections in free speech^124^. Scherer et al. demonstrated that the voices of participants with moderate to severe depression are tenser than those of healthy participants^118^. Alghowinem et al. proposed that spontaneous speech leads to better results for most features than reading speech and that the first few seconds of speech perform better than the entire recording^125^. Another interesting approach to emotion elicitation is to use virtual agents for interviews, which can reduce data collection costs and can be less stressful for participants when discussing their symptoms. Multiple articles have reported successes in developing virtual interviewers^126–128^, and the widely used AVEC challenges have also adopted virtual interviewers.

**Table 7:** A comparative study of different speech-eliciting tasks.

Task and examples		Advantages
Constrained	Repeating “PATAKA”^187,188^	Capture speech sequencing; a proxy for lung capacity
	Sustained vowel^189^	Measure muscle weakness and aspects of move control
	Counting^190^	Counting from 1 to 10 allows mroe control over acoustic patterns
	Reading	
	• The “Nordwind” passage^191,192^	Paragraphs frequently used in the gathering of depression-related speech
	• Rainbow passage^189^	Includes all the sounds used in English and reflects normal speech patterns
	• Emotion-evoking movie clips^193^	Greater ability to regulate emotions that are provoked
Free speech	Monologue	
	• Describing, memory recalling^194^	More spontaneous than reading speech
	Dialogue	
	• Semi-structured interviews^120^	Frequently used in medical facilities
	• Phone conversations^195^	Only the interviewee is recorded; no need to identify the speaker

### Diarization of speech segments in interview recordings: methods and considerations

It is common practice to separate the speech segments of the participants from interview recordings to train depression prediction models. This process is commonly referred to as diarization. The participant’s speech can be extracted using a microphone next to each speaker. If participants have headsets with lapel microphones during the interview, their voiced sections can be easily extracted, which may make some participants uncomfortable. Desk microphones can also be used, but they can introduce confounds because they are not targeted and make it difficult to separate participants’ speech in the data processing. We suggest using two desk microphones next to each speaker with a sound barrier between them. It is important to record all metadata after the interview in a separate spreadsheet, such as participant ID, group, task, and other demographic information.

### Ensuring privacy in interview recordings

Clinicians should obtain verbal or written consent from the participants before interviewing. Participants must be informed that their interview recordings and demographic information may be distributed, pre-processed, and used for training machine-learning models for academic research purposes. Even if participants grant permission for their data to be further processed, researchers must minimize the risk of data leakage because the interview audio (or video) recordings may contain sensitive information. To address this, researchers can share only the automated extracted speech and facial features rather than the raw interview recordings. If hackers were to gain access to the interview data, it would be impossible to reconstruct the original interview recordings using only the automated extracted features. Additionally, researchers can train the depression prediction model in real-time or use bone conduction microphones, which only record acoustic features without speech content^129^, but this limits researchers to training semantic models. Edge computing can also be a solution to improve privacy by allowing computation to be performed on the participants’ devices, with only the trained models being returned to the researchers, not the data.

### Review of machine-learning models

We believe that a well-trained depression prediction model can accurately detect the disorder in a randomly selected individual, regardless of the environment in which the individual is being interviewed. The participant may be of a different age and use a different language or accent, but the depression prediction model should still be able to generalize and be robust to these environmental factors. However, most previous studies have only been designed to detect mental disorders in new individuals collected in similar settings. Our suggestions for future studies to improve robustness and generalization are as follows.

### Data preprocessing

Automatic speech recognition (ASR) can transcribe speech into transcripts for training semantic-based depression prediction models. ASR can also filter unvoiced sections and noise in the interview audio (or video) recordings. In most in-person interviews, two speakers are present, and the clinicians’ segments can be discarded if the ASR system includes automatic diarization. To prevent overfitting, techniques like dimensionality reduction or feature selection should be applied to the training and test sets during preprocessing. This will help ensure that the model is not overly influenced by the specific characteristics of the training data and can be generalized to new data.

### Automated feature extraction

The most commonly used automated feature extraction tools in the studies we reviewed were openSMILE, COVAREP, pyAudioAnalysis, and openEAR. As shown in Table 8, some features were found to be predictive in multiple studies. To ensure the deep learning models converge, it is recommended to standardize or normalize features as they may be in different scales. Before training the model, we recommend performing exploratory data analysis or visualization to better understand how these features characterize mental disorders. This can help inform the selection and preprocessing of features, as well as the design of the model.

**Table 8:** An overview of acoustic features. for more details, see the cooperative voice analysis repository (COVAREP).

Acoustic feature	Description
Source features	Features reflecting airflow from the lungs through the glottis (i.e., glottal features) or vocal fold vibrations (i.e., voice quality features), which is the sound source later filtered by the vocal tract following the source-filter theory of speech production.
Jitter (%)	Deviations in the consecutive lengths of the *f*_0_ period, which suggests irregular and uneven vocal fold vibrations.
Shimmer (%)	The variation in the peak amplitudes of consecutive *f*_0_ periods, which implies unevenness in voice loudness.
Tremor (Hz)	The number of occurrences of the most powerful low-frequency fundamental frequency-modulating element within a defined examination range.
Harmonics-to-noise ratio (HNR) (dB)	Ratio between *f*_0_ and noise components, which indirectly correlates with perceived aspiration.
Frequency disturbance ratio (FDR) (%)	The average relative value of the frequency variation over 5 to 5 cycles (calculated using an average of five data points).
Amplitude disturbance ratio (ADR) (%)	Relative mean amplitude value over a set of windows.
Quasi-open quotient	Ratio of the vocal folds opening time. Functional dysphonias often reduce QOQ range.
Normalized amplitude quotient (NAQ)	A measurement that compares the amplitude between the highest and lowest points of the differentiated flow glottogram to the amplitude of the negative peak and normalizing it with respect to the period time. It can be used as an approximation of glottal adduction.
Peak slope	Slope of the regression line that is fit to log10 of the maxima of each frame.
Filter features	The resonant properties of the vocal and nasal tracts filter the sound source from the vocal folds: the filter attenuates certain frequencies and strengthens others by the shape of the vocal and nasal tracts.
*F*_1_ mean (Hz)	First peak in the spectrum that results from a resonance of the human vocal tract.
*F*_2_ mean (Hz)	Second peak in the spectrum that results from a resonance of the human vocal tract.
*F*_1_ variability (Hz)	Measures of dispersion of *F*_1_ (variance, standard deviation).
*F*_2_ variability (Hz)	Measures of dispersion of *F*_2_ (variance, standard deviation).
*F*_1_ range (Hz)	Difference between the lowest and highest *F*_1_ values.
Vowel space	*F*_1_ and *F*_2_ 2D space for the vowels.
Linear predictive coding (LPC) coefficients	Coefficients that best predict the values of the next time point of the audio signal using the values from the previous n time points, which is used to reconstruct filter properties.
Spectral features	Features characterizing the frequency distribution of the speech signal at a particular moment in time.
Mel-frequency cepstral coefficients (MFCCs)	The coefficients derived by analyzing the Mel-spectrum of the log-magnitude of an audio segment.
Prosodic features	Changes over longer segments of time, which is perceived in the rhythm, stress, and intonation of speech.
*f*_0_ mean (Hz)	Fundamental frequency: lowest frequency of the speech signal, perceived as pitch (mean, median).
*f*_0_ variability (Hz)	Measures of dispersion of *f*_0_ (variance, standard deviation).
*f*_0_ range (Hz)	Difference between the lowest and highest *f*_0_.
Intensity (dB)	Defined as the acoustic intensity (i.e., power carried by sound per unit area in a direction perpendicular to that area in decibels relative to a reference value, perceived as loudness).
Intensity variability (dB)	Measures of dispersion of intensity (variance, standard deviation).
Energy velocity	Measured as the mean-squared central difference across frames and may correlate with motor coordination.
Maximum phonation time (s)	The mean of three attempts of the following measure is taken: the maximum time during which phonation of a vowel is sustained as long as possible with an upright position, deep breath, and a comfortable pitch and loudness.
Speech rate	Number of speech utterances per second over the duration of the speech sample (including pauses).
Articulation rate	Number of speech units per second throughout the speech sample (excluding pauses).
Time talking (s)	Sum of the duration of all speech segments.
Utterance duration mean (s)	Mean duration of utterance length.
Pause duration mean (s)	Mean duration of pause length.
Pause variability (s)	Measures of dispersion of pause duration (variance, standard deviation).
Pause rate (s)	Total length of pauses divided by the total length of speech (including pauses).
Pause total (s)	Total duration of pauses.

### Evaluate models with small datasets: bootstrapping and K-fold cross-validation

To avoid overfitting the model on the test set, we typically evaluate the trained model on the held-out test set only once. However, when training a model for predicting depression using a small dataset (e.g., around 100 data points), which is commonly seen in the medical field, a 20% held-out test set or K-fold cross-validation can decrease the number of samples available for training. In addition, a small test set is unlikely to represent the entire population accurately. As a result, we suggest using repeated bootstrapping to evaluate the depression prediction model, which provides a distribution of performance metrics with mean and standard deviation. However, given the computational complexity of deep learning models, the bootstrapping method may not be feasible. When working with a small dataset, K-fold cross-validation can be a viable alternative to bootstrapping, as deep learning models tend to need a large number of data points which reduces the need for bootstrapping.

### Evaluating the performance of depression prediction: best practices and considerations

Performing better than chance does not indicate the model learned from the training data, and the resulting metrics must be generalizable and statistically significant for clinical use. Aloshban et al. demonstrated that their accuracy is always better than chance to a statistically significant extent^130^. However, to further prove generalizability, we suggest performing a permutation test in future works, where models are trained on permutated labels to evaluate the model’s performance based on mistaken labels, which is often better than chance. A statistical test can then determine if the difference between the permuted and non-permuted scores is statistically significant. On the other hand, clinical datasets can be imbalanced, with a greater number of healthy cases compared to the population of individuals with depression. In this case, using the accuracy of the classification model as the sole metric to evaluate its performance may not be objective since it will be biased towards predicting every sample as negative. To evaluate model performance more objectively, metrics such as the *F*_1_ score, precision, recall, and area under the curve (AUC) should be considered. These metrics account for class imbalance and provide a more balanced view of model performance. Saito et al. have shown that the precision-recall curve is more useful than the receiver operating characteristic curve when evaluating binary classifiers on imbalanced datasets^131^. In addition to the precision-recall curve, metrics such as root mean square error (RMSE), mean square error (MSE), and the coefficient of determination (*r*^2^) are commonly used to evaluate the performance of regression models for predicting depression scores. In the AVEC competition, the performance of baseline models was evaluated using the concordance correlation coefficient (CCC), which takes into account changes in scale and includes measures of both precision and accuracy^132^. It is generally helpful for other researchers to see a range of metrics when evaluating the performance of a model, as this allows for more objective comparison. A model’s performance must be generalizable and statistically significant to be truly useful in a clinical setting.

### Explainable depression detection model

Recent evidence suggests that individuals may lack trust in black box models and that these models may cause harm in high-stakes decision-making processes^133–135^. As a result, researchers are exploring ways to explain the decision-making processes of algorithms better^136,137^ through publications and software packages implement explainable machine-learning models^138,139^. By providing explanations of a model’s feature contributions, clinicians can gain a better understanding of depression and improve the model itself. Lundberg et al. proposed an additive method for evaluating feature contributions, which calculates the difference in the model’s output when a given feature is considered versus not considered^139^. This can provide valuable insights into the factors influencing the model’s predictions. Once high-impact features have been identified, we can retrain the model using only these features to evaluate their performance. In some of the reviewed articles, we observed that while the studies presented excellent feature engineering for distinguishing between groups, they lacked quantitative analysis to support their findings. In addition, we suggest linking changes in the automated extraction of features to mental disorder symptoms to provide a more comprehensive view of the model’s performance. By combining these approaches, we can better understand the factors influencing the model’s predictions and improve its performance. On the other hand, there are arguments on whether a complex, hard-to-explain model with good performance should be discarded in favor of a simpler, easy-to-interpret but lower-performing model^140^. From our perspective, since complex but high-sensitivity examination methods would not surpass the use of less sensitive models, these models would not be abandoned if they are proven effective in clinical trials. Thus, validating and explaining complex models will be important in this field.

### Ensuring reproducibility in automated depression detection

Reproducibility is a critical issue in machine learning, particularly when artificial intelligence is applied to healthcare^141,142^. One obstacle to reproducing previous studies is that clinical datasets are not always available for redistribution. As we mentioned in Section Ensuring privacy in interview recordings, automated extracted features can be shared without violating privacy concerns. However, sharing the code used for training and evaluating the model is also important. Even when the code and data are publicly available, other researchers may still have difficulty reproducing the results due to differences in the software environment. To address this issue, we suggest that researchers use containers, such as Docker, which include the data, code, and environment in one package that can be easily redistributed. This will make it easier for other researchers to reproduce the results, ultimately accelerating the advancement of automated depression detection.

### Evaluating models from competitions in automated depression diagnosis

About 39% of the studies included in this review were developed during or after the AVEC. Some of these studies introduced innovative approaches to feature engineering and model architecture. However, since teams were allowed to submit their models multiple times, overfitting the test set is a risk. The test set provided in the AVEC competition is relatively small, which means that the state-of-the-art model can outperform the second-best model by chance due to overfitting. Therefore, we cannot simply assume that a model that performs slightly better on the test set will also generalize well to new data. We believe that multiple comparison corrections should be applied to evaluate the performance of the models, and simpler models should be prioritized^143^. This will help to ensure that the results are more reliable and can be more confidently applied in a clinical setting.

Due to the limited number of studies we were able to review and include in this review, we only searched for keywords in the titles of articles rather than in other sections. The screening process of the articles involved reading the title and abstract. Only articles that were relevant to using machine learning to detect depression and had “machine learning" or related terms in the title were included, and the others were excluded. Our study may not have captured all relevant articles on this topic, and other studies not focused specifically on automated depression diagnosis using machine-learning methods may have been missed.

### Future work

#### Cross-cultural generalization

While machine-learning models are optimized to work well on the training data, it is not always clear how well they will generalize to new sample sets where different ages, languages, socioeconomic and education levels are reported. Alghowinem et al. developed a cross-cultural depression recognition model and evaluated it on datasets in English and German^144^. Some previous studies have also investigated the effects of different smartphones on the quality of acoustic features, which can impact the accuracy of depression prediction^145,146^. Mitra et al. examined the effects of noise and reverberation on depression detection using speech and found that spontaneous speech performed better than read speech^147^. These studies highlight the importance of considering factors that may affect the performance of machine-learning models for automated depression diagnosis.

#### Ethical considerations in automated depression detection: addressing risks and ensuring responsible use

Automated depression detection can benefit society by reducing the workload of the healthcare system, preventing suicidal or self-harm behaviors, and enabling law enforcement authorities to track abnormal behaviors. However, the use of automated depression detection also raises some ethical concerns. For example, insurance companies and employers may use the results to evaluate candidates without their knowledge or consent and reject them if a mental disorder is present or likely to develop in the future. In addition, it can be difficult for individuals to fully understand the implications of consent forms, which can further complicate the ethical considerations surrounding automated depression detection^148^. To ensure that automated depression detection is used ethically in clinical settings, researchers should provide clear and understandable explanations of how the collected data will be used. Participants should also have the right to revoke permission to use their data at any time. Like other developing technologies, these systems may be vulnerable to abuse and have unexpected side effects. As researchers, engineers, and clinicians, it is our responsibility to educate the public and policymakers about the potential benefits and harms of automated depression detection to both prevent abuse and further advance these techniques, which have the potential to help many people.

#### Leveraging machine learning for advancing psychiatry

With this review paper, we aim to demonstrate the potential for psychiatry to benefit from advances in machine learning. Many individuals have difficulty accessing qualified mental healthcare or may be hesitant to seek psychotherapy due to stigmatization^149^. Automated depression detection models can provide an accessible and efficient method for early screening, which can help individuals determining that they may need professional healthcare. In addition, psychiatric visits often include interviews that can be recorded in video or audio format, which provides a wealth of data that can be used to associate mental health assessments with acoustic, semantic, and facial features. By following the guidelines outlined in this paper for collecting and analyzing this data, we hope to enable new collaborations between clinicians and machine-learning engineers to advance our understanding of mental health disorders.

## Conclusion

We reviewed 264 studies that measure acoustic, semantic, and facial landmark features to distinguish between individuals with and without mental health disorders using either null hypothesis testing or predictive machine-learning models. Our synthesis includes significant and nonsignificant features across audio, text, and facial modalities, as well as those correlated with the severity of depression. We also provide guidelines on collecting data, preventing confounding factors, protecting privacy, selecting speech-eliciting tasks, and improving machine-learning model generalizability and reproducibility. We also found a few studies have been conducted on post-traumatic stress disorder, bipolar disorder, and postpartum depression, thanks to open-access research datasets provided by the AVEC and DAIC. Competitions provide a useful framework for comparing innovations under the same conditions, such as using the same dataset and metrics. This approach enables researchers to evaluate the model’s performance using a held-out test set and estimate overfitting; however, overfitting is still a concern in such competitions. In addition, these competitions make it possible for future studies to be conducted using the same dataset, which facilitates comparisons and helps advance the field. Based on their proven effectiveness, we encourage the collection of open datasets, particularly distributing datasets through competitions. These are highly productive in advancing research in various fields. While productivity is important, reproducibility is also critical. Since the studies in this review involve building computational models, the associated data and code should be shared, ideally through containers. This allows others to test the claims made by these studies and contribute to the development of these models in a collaborative manner. Moreover, conducting more research on multiple datasets may help enhance the models’ generalizability and reconcile conflicting results regarding crucial and predictive features. This approach could lead to more robust and reliable conclusions about the nature of these disorders and their diagnosis and treatment. Using multimodality features to train machine-learning models holds promise for enhancing mental health evaluations and treatment. This approach aligns with the principles of preventive and personalized diagnosis and treatment and could lead to better outcomes for individuals with mental health conditions.

## Supplementary information

Reporting Summary

Checklist item

## Data Availability

The data supporting this review paper are openly available and can be accessed at the following URLs: https://bit.ly/3DBQtZk; https://bit.ly/43Q6Yvy; https://bit.ly/44IKaPv.

## Code availability

The code associated with this review paper is openly available and can be accessed at the following GitHub repository: https://github.com/uofabinarylab/ADDReview.
